# Legal Notes for Institutional Workers

**Published:** 1912-08-31

**Authors:** 


### LEGAL NOTES FOR INSTITUTIONAL WORKERS

(The Editor will be pleased to answer Legal queries in this column.)

### Danger from Aerial Convection.

No serious attempt to interfere with a hospital on the ground that it is a nuisance has been made since 1904. In that year it was alleged by residents in the neighbourhood of Nottingham that the erection of a smallpox hospital was *per se* a danger to health and a public and private nuisance. Scientific evidence was offered as to the aerial convection of this disease for a considerable distance. An injunction was accordingly sought to restrain the erection. It was held that, to justify an injunction, it was necessary, not merely to show that the hospital would abridge liberty or create anxiety, but that it would prove an actionable nuisance, and that the plaintiffs having failed to show this, the action failed.

### Smallpox Hospitals and the Law.

It was reserved to an Irish Lord Justice to state the law of nuisance as it is applicable to smallpox isolation hospitals: "It seems probable," he said, "that the dread of smallpox is to a great extent the result of tradition. That scourge of the 18th century retains its terrors for those who do not realise that it has been deprived of most of its dangers. Vaccination is not only a prevention, but it also modifies the disease. . . . In applying the maxim *Sic utere tuo ut alienum non lædas*, the duty of reasonable precaution for one's own protection is not to be ignored. An isolation hospital reduces the risk for every inhabitant of the district. It is, in fact, a necessity, and though the individual must be protected, the public advantage should not be forbidden unless the danger and injury to the individual are clearly proved."

### Hospital Fire Brigades.

It is necessary for the safety of the inmates of every large institution that some members of the staff be trained to act as a fire brigade. Like the American cowboy's revolver, the brigade "is not often wanted, but when it is wanted it is wanted mighty badly." As the use of a fire-escape is somewhat dangerous, care should be taken that the institution is insured in respect of possible accidents to members of the brigade. The porter acting as fireman would clearly be the victim of an accident arising out of and in the course of his employment if he were knocked down by a falling ladder or burnt. The legal position of convalescent patients and others who would naturally assist in extinguishing a fire is not so easy to determine; but it would, doubtless, be possible to frame an insurance policy which would cover the entire risk of accident to any person assisting to extinguish the fire.

### Milk Containing a Preservative.

Inasmuch as milk forms the staple diet of countless hospital patients it is important that managers should know something of the law with regard to its sale. The sale of milk with a preservative is legal provided the fact that a preservative is present is drawn to the attention of the purchaser. Nor does it matter that the exact amount of preservative used is inaccurately stated. In the recent case of *Williams* v. *Friend*, 76 J. P. 301, the appellant, the proprietor of a dairy, sold to an assistant of the respondent, who was an inspector under the Sale of Food and Drugs Acts, a quantity of cream. Before being supplied with the cream the purchaser read a notice stating that all cream sold at the establishment contained boron preservative (not exceeding half of 1 per cent.) to keep it sweet and wholesome. This percentage was equal to 35 grains to the pound. The analysis showed that it contained 33.838 grains of boric acid per pound. On a prosecution against the appellant under section 6 of the Sale of Food and Drugs Act, 1875, for selling to the prejudice of the purchaser an article not of the nature, substance, and quality demanded, the justices found that the article was not of the nature, substance, and quality demanded, that the sale was to the prejudice of the purchaser, and that the article was injurious to health, and they convicted the appellant. The Divisional Court, however, has held that the conviction must be quashed, as the purchaser had notice that the article was mixed with something else, and therefore there was no evidence that there was a sale to his prejudice.

